# Role of a lipid metabolism-related lncRNA signature in risk stratification and immune microenvironment for colon cancer

**DOI:** 10.1186/s12920-022-01369-8

**Published:** 2022-10-21

**Authors:** Yaobin Lin, Yu Xiao, Shan Liu, Liang Hong, Lingdong Shao, Junxin Wu

**Affiliations:** 1 Department of Radiation Oncology, Clinical Oncology School of Fujian Medical University, Fujian Cancer Hospital, 420 Fuma Rd, Jin’an District, Fuzhou, 350014 Fujian China; 2 Department of Hematology-Oncology, Fujian Children’s Hospital, Fuzhou, 350000 China

**Keywords:** Colon cancer, Lipid metabolism, Long non-coding RNAs, Prognosis, Tumor microenvironment

## Abstract

**Background:**

Energy metabolism disorder, especially lipid metabolism disorder, is an important biological characteristic of colon cancer. This research sought to examine the association between lipid metabolism-related long non-coding RNAs (lncRNAs) and prognoses among colon cancer patients.

**Methods:**

The transcriptome profile and clinical data of patients with colon cancer were retrieved from The Cancer Genome Atlas database. Using consensus clustering, cases were divided into two clusters and Kaplan–Meier analysis was executed to analyze differences in their prognoses. The gene set enrichment analysis (GSEA) was used to discover biological processes and signaling pathways. A lipid metabolism-related lncRNA prognostic model (lipid metabolism-LncRM) was created utilizing the least absolute shrinkage and selection operator (LASSO) regression. The tumor microenvironment was evaluated on the basis of the composition of immune and stromal cells.

**Results:**

The patients in Cluster 2 were found to have a better prognosis and higher expression of programmed cell death 1 (PD-1) and programmed cell death ligand 1 (PD-L1) relative to Cluster 1. The results of GSEA showed the enrichment of energy metabolism pathways in Cluster 2. LASSO regression was used to identify the five LncRNAs that were shown to be most substantially linked to patient prognosis. These were NSMCE1-DT, LINC02084, MYOSLID, LINC02428, and MRPS9-AS1. Receiver operating characteristic (ROC) curves and survival analysis illustrated that the lipid metabolism-LncRM had a significant prognostic value. Further analysis showed that high- and low-risk groups were significantly different in terms of clinical characteristics and immune cells infiltration.

**Conclusions:**

Lipid metabolism-related lncRNAs could predict the prognoses and tumor microenvironment of colon cancer and might be important biomarkers relevant to immunotherapy.

**Supplementary Information:**

The online version contains supplementary material available at 10.1186/s12920-022-01369-8.

## Introduction

Colon cancer is the most prevalent malignant illness of the gastrointestinal tract [1], whereby the incidence and mortality rates account for 6.0% and 5.8% of all cancers [2, 3]. At present, the treatment modalities include chemotherapy, radiotherapy, endoscopic and surgical local excision, targeted therapy, and immunotherapy. These treatment regimens can improve the patients' 5-year overall survival (OS) rate is 60–70% [4]. Tumors are often heterogeneous. Characterizing tumors into different subtypes and designing individualized treatment strategies according to their biological characteristics can improve cancer control rates. Hence, it is necessary to recognize new markers for anticipating the prognoses of colon cancer patients and screening those who may stand to substantially benefit from immunotherapy.

Metabolic reprogramming has emerged as a prominent feature of cancer; it promotes tumor cell proliferation and survival [5], whereby lipid metabolism disorder is a prominent metabolic change in tumors. Active fat metabolism often occurs in patients with aggressive metastatic colon cancer [6]. Lipid metabolism disorder can lead to the alteration of the expression and activity of lipid metabolic enzymes due to abnormally activated carcinogenic signal pathway, redistribution of lipid metabolism in cancer cells, along with enhanced rapid proliferation, migration, and invasion of cancer. In addition, Gharib et al. [7] report that microRNA-497-5p facilitates colon cancer cell death induced by starvation via targeting acyl-CoA synthase-5 and regulating lipid metabolism. Nevertheless, as far as we know, there are no studies on the association of lipid metabolism, long non-coding RNAs (lncRNAs) with colon cancer advancement.

lncRNAs are a widely present type of RNA molecule with a length exceeding 200 nucleotides but no ability for protein-coding [8]. According to recent research, lncRNAs may influence tumor growth by modifying fatty acid metabolism [9–11]. Because it binds to the arginine residues of the hnRNPA1-RGG motif, the lncRNA HOXB-AS3 has the potential to significantly suppress glucose metabolic reprogramming in colon cancer cells [12]. This study aimed at evaluating whether targeting both lipid metabolism-related genes and lncRNAs may be utilized as novel therapeutic targets against colon cancer.

Herein, the data on colon cancer were extracted from the cancer genome atlas (TCGA) database and we investigated the association of lipid metabolism-related lncRNAs with clinical characteristics and immunotherapy. Based on lipid metabolism-related lncRNAs, the Least Absolute Shrinkage Selection Operator (LASSO) was utilized to construct a prognostic risk stratification model. In addition, we also evaluated the immune statuses of patients in different risk groups.

## Methods

### Data collection

The study flow is illustrated in Additional file 1: Figure S1. We acquired the RNA-seq and clinical data of patients with colon cancer from the TCGA database (https://portal.gdc.cancer.gov/); a total of 4,234 lncRNAs were obtained. We extracted the data of 514 samples, of which 473 were from tumor tissues and the remaining were from adjacent normal tissues. The clinical information was available for 446 cases which also had complete RNA-seq data. Using the Molecular Signatures Database v7.0, 189 genes related to lipid metabolism were identified (Additional file 2: Table S1) [13, 14]. Ethics approval and informed consent were not required in this study since the data were obtained from public databases. The study was conducted in accordance with the declaration of Helsinki.

### Identification of lipid metabolism-related lncRNAs

The differential expressions of lncRNAs between cancer and normal specimens were compared utilizing the *R* package, “limma”. Spearman correlation analysis was carried out to screen lipid metabolism-related lncRNAs correlated with at least one lipid metabolism gene (| Pearson *R* |> 0.6 and *p* < 0.001). The co-expression network of lipid metabolism-related genes and lncRNAs was created utilizing the R package, “igraph”. For the purpose of screening lncRNAs that were correlated with OS, a univariable Cox regression was utilized.

### Analysis of functional enrichment

On the basis of the Kyoto Encyclopedia of Genes and Genomes (KEGG) pathways [15–17], the Gene Set Enrichment Analysis (GSEA) (https://www.gsea-msigdb.org/gsea/index.jsp) was employed to identify biological functions and signaling pathways in different clusters. Enriched genes were defined according to the threshold values of *p* < 0.05 and false discovery rate < 25%.

### Evaluation of cell compositions in tumor microenvironment

Using ESTIMATE, we assessed the immune and stromal cells in malignant tissues, computed stromal and immune scores, and predicted the cellular infiltration in the tumor microenvironment (TME) [18]. StromalScore refers to the infiltration level of stromal cells in the tumor tissue; ImmunoScore represents the infiltration level of immune cells; and ESTIMATEScore is an indicator that combines StromalScore with ImmunoScore to infer tumor purity. By employing CIBERSORT (https://cibersort.stanford.edu/), we computed the proportion of 22 distinct immune cell infiltrates. Based on the R package “ImmuneSubtypeClassifier” and “TCGAbiolinks”, samples were divided into different immune subtypes and molecular subtypes [19, 20].

### Construction and verification of the risk signature

The LASSO regression analysis was carried out with the help of the R package “glmnet” to create a prognostic model based on lipid metabolism-related lncRNAs (lipid metabolism-LncRM). The calculation for the model was as follows:

$$Risk{\text{ score = }}\sum\limits_{i = 1}^{n} {coefi*xi}$$

We calculated the risk scores according to the level of each lncRNA expression and the matching regression coefficient. The estimation of the risk score was achieved according to the equation below: Risk score = (0.7641 × NSMCE1-DT) + (0.1906 × LINC02084) + (1.2843 × MYOSLID) + (0.2206 × LINC02428) + (0.1157 × MRPS9-AS1). Patients were categorized at random into training (*n* = 224) and testing subsets (*n* = 222) with the help of the R package, “caret”, and assigned to high- and low-risk groups on the basis of the mean risk score obtained in the training set. Univariate and multivariate Cox regression analyses were employed to examine the independent prognostic values of risk scores.

### Statistical analysis

The R statistical program version 3.6.1. was utilized to execute all analyses of statistical data. The survival in subgroups was computed utilizing the Kaplan–Meier curves. We used the “survivalROC” package for receiver operating characteristic (ROC) curve analysis to examine the prediction accuracy. The differential expressions of lncRNAs between cancer and normal samples were compared utilizing the Wilcoxon signed-rank test. Differences in qualitative variables were examined using the *χ*^2^ test. The relationship among the quantitative variables was assessed utilizing Spearman's correlation analysis. *P* < 0.05 was established as a determinant of statistical significance.

## Results

### Selection of lipid metabolism-related lncRNAs

**Fig. 1:**
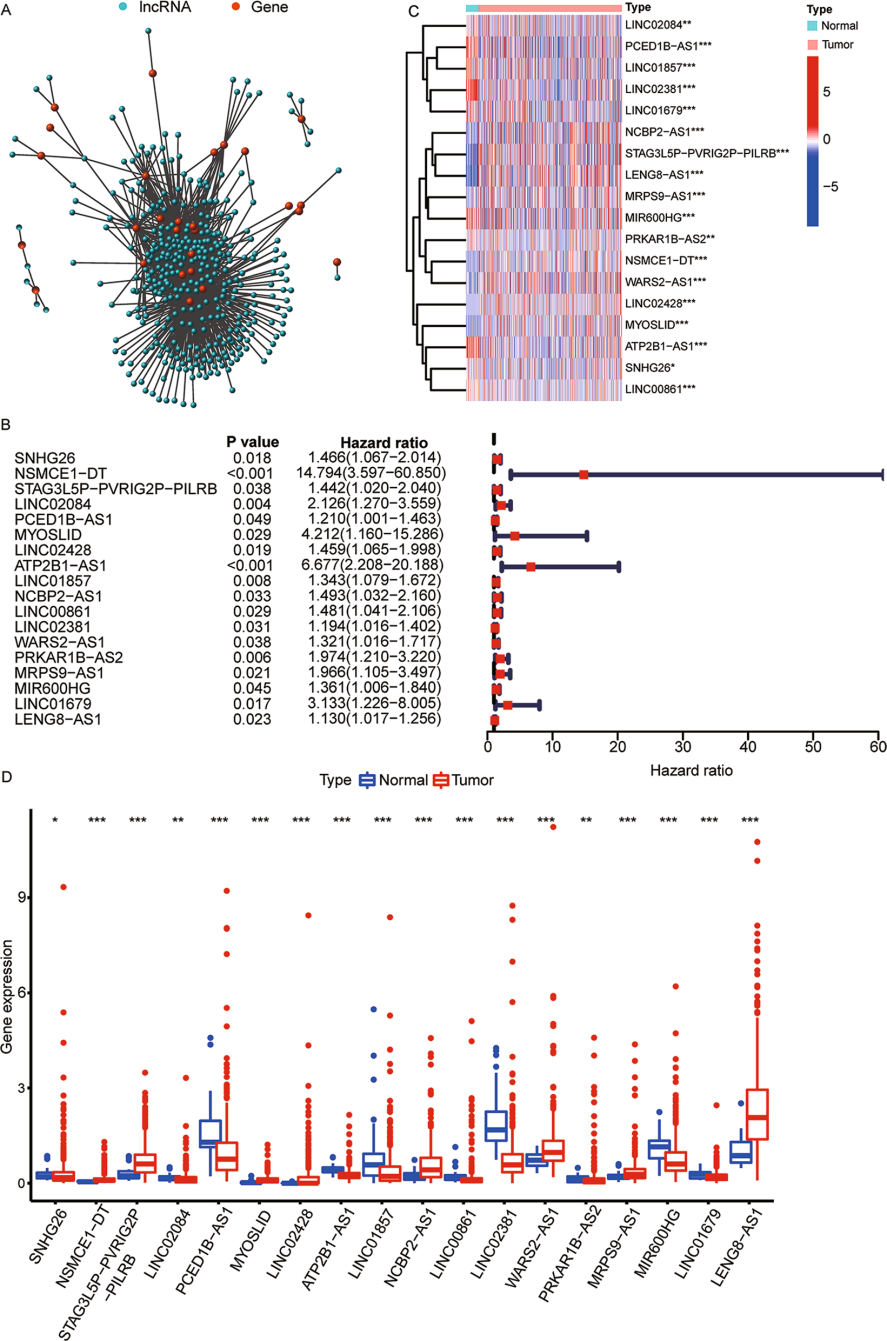
Identification of lipid metabolism-related lncRNAs in colon cancer. **A** The co-expression network of lipid metabolism-related genes and lncRNAs. **B** Forest plot based on the univariate Cox regression analysis for OS. **C** Heatmap and **D** box plot for the differential expressions of 18 lipid metabolism-related lncRNAs between tumor and normal tissues. lncRNAs, long non-coding RNAs; OS, overall survival. *p < 0.05, **p < 0.01, and ***p < 0.001

The clinical information of 446 colon cancer patients in TCGA is shown in Table 1. In total, 27 genes and 349 lncRNAs related to lipid metabolism were screened using the Spearman correlation analysis (Fig. 1A). Next, based on the clinical data, we filtered out 18 lipid metabolism-related lncRNAs significantly related to prognosis. The forest plot shows the hazards ratio and 95% confidence intervals (CIs) of the 18 lipid metabolism-related lncRNAs (all *p* < 0.05, Fig. 1B). Figures 1C and 1D demonstrate that in tumor tissues, the expressions of 8 lipid metabolism-related prognostic lncRNAs (NSMCE1 − DT, STAG3L5P − PVRIG2P − PILRB, MYOSLID, LINC02428, NCBP2 − AS1, WARS2 − AS1, MRPS9 − AS1, and LENG8 − AS1) were upregulated, whereas that of the remaining 10 lncRNAs (SNHG26, LINC02084, PCED1B − AS1, ATP2B1 − AS1, LINC01857, LINC00861, LINC02381, PRKAR1B − AS2, MIR600HG, and LINC01679) were markedly downregulated (all *p* < 0.05).

**Table 1:** Clinical characteristics of patients with colon cancer

Character	Number (%)
Total	446 (100)
Age (years)	
< = 65	183 (41.0)
> 65	263 (59.0)
Sex	
Male	234 (52.5)
Female	212 (47.5)
T stage	
T1-2	87 (19.5)
T3-4	359 (80.5)
N stage	
N0	265 (59.4)
N1-2	181 (40.6)
M stage	
M0	377 (84.5)
M1	61 (13.7)
Unknown	8 (1.8)
Total stage	
Stage I-II	253 (56.7)
Stage III-IV	185 (41.5)
Unknown	8 (1.8)

### Definition of colon cancer subtypes according to lipid metabolism-related lncRNAs

**Fig. 2:**
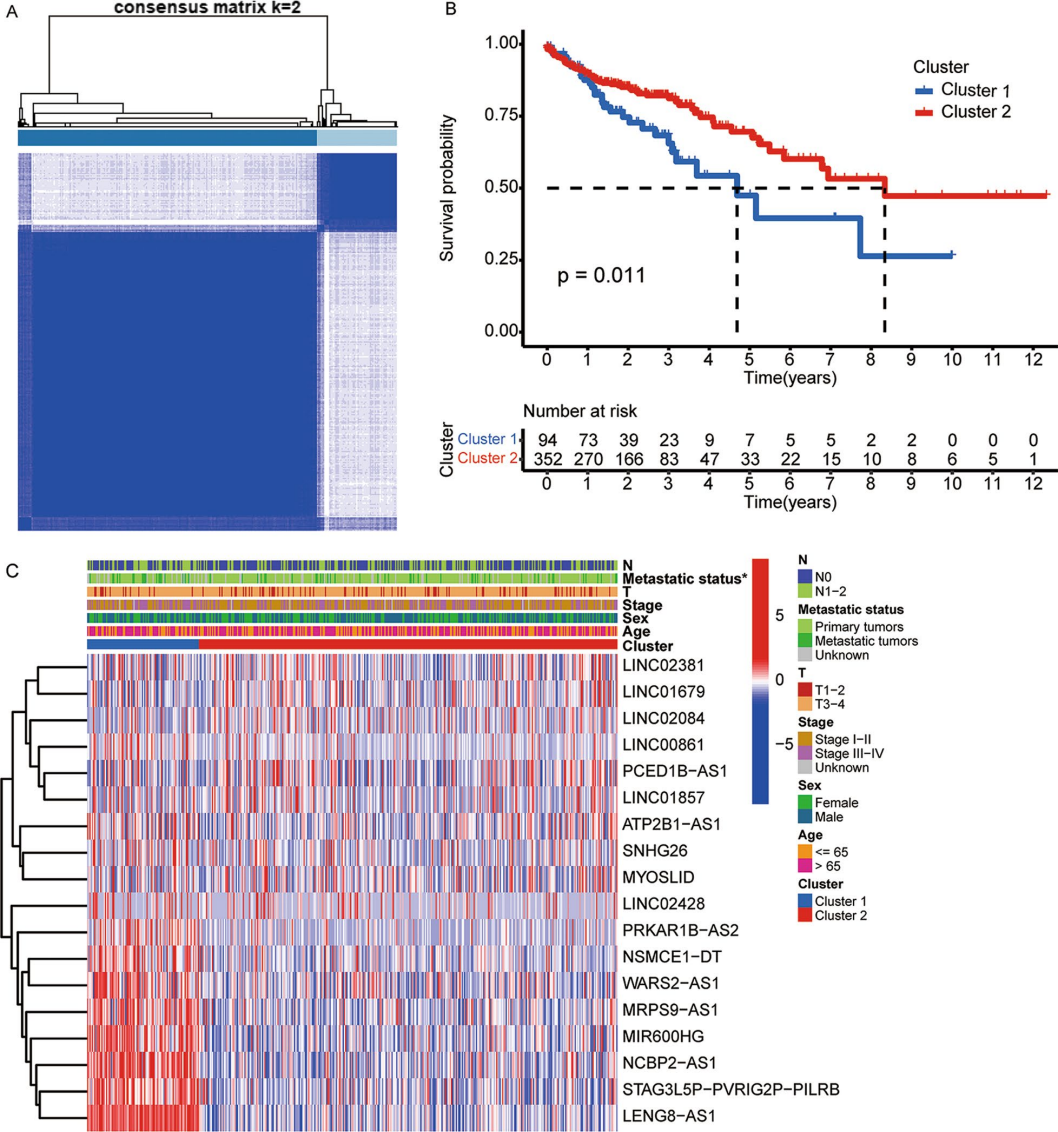
Identification of tumor subtypes based on the prognostic lipid metabolism-related lncRNAs. **A** Consensus clustering matrix at *k* = 2. **B** Kaplan–Meier plot for patients in Clusters 1 and 2. **C** Heatmap of 18 prognostic lipid metabolism-related lncRNAs and clinicopathological features in Clusters 1 and 2. *p < 0.05

The consensus clustering analysis based on the lipid metabolism-related lncRNAs was used to examine the molecular subtypes of colon cancer. At *k* = 2, the consensus matrix heatmap showed sharp and crisp boundaries (Fig. 2A; Additional file 3: Figure S2), which indicated that the samples could be divided into stable and robust clusters. Therefore, all data were assigned to two clusters. Patients in Cluster 2 showed significantly longer OS relative to those in Cluster 1 (*p* = 0.011, Fig. 2B). Association analysis of subgroups and clinical features presented that only the distribution of the metastatic status was different between clusters (*p* < 0.05, Fig. 2C). This suggested that the difference in survival between the two clusters might be related to their different risk of metastasis.

**Fig. 3:**
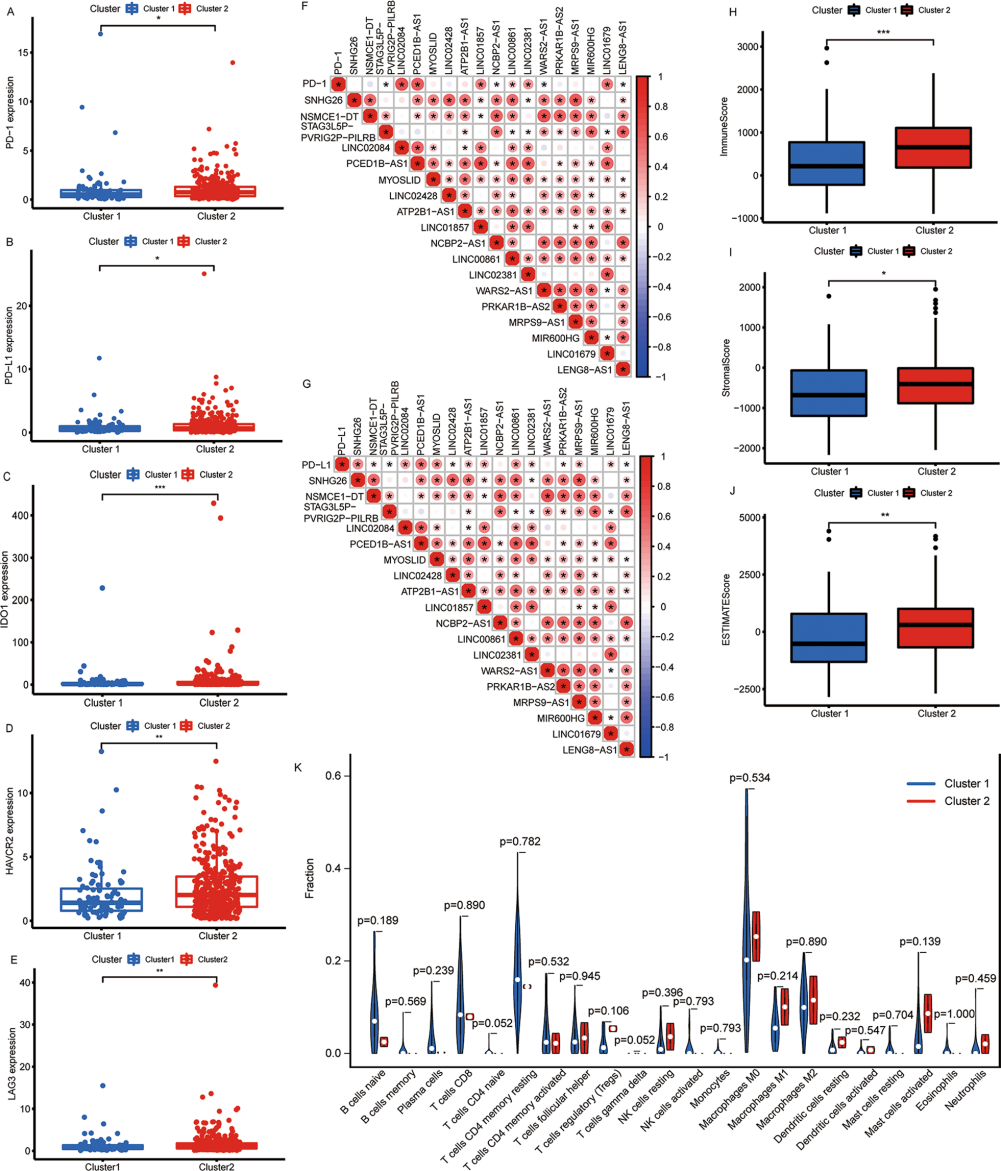
Relationship between the Clusters and immune characteristics. The expression of immune checkpoints **A** PD-1, **B** PD-L1, **C** IDO1, **D** HAVCR2, and **E** LAG3 in Clusters 1 and 2. **F**-**G** Correlations among the 18 lipid metabolism-related lncRNAs and immune checkpoints. Differences in **H** immune, **I** stromal, and **J** estimate scores in Clusters 1 and 2. **K** Differences in the abundances of 22 immune cell types between Clusters 1 and 2. PD-1, programmed cell death 1; PD-L1, programmed cell death ligand 1; IDO1, indoleamine 2,3-dioxygenase 1; HAVCR2, hepatitis A virus cellular receptor 2; LAG3, lymphocyte activating gene 3. *p < 0.05, **p < 0.01, and ***p < 0.001

Next, the relationship among lncRNAs related to lipid metabolism and immune characteristics in colon cancer was investigated. First, the levels of expression of immune checkpoints (programmed cell death 1 [PD-1], programmed cell death ligand 1 [PD-L1], cytotoxic T lymphocyte antigen-4 [CTLA-4], indoleamine 2,3-dioxygenase 1 [IDO1], indoleamine 2,3-dioxygenase 2 [IDO2], hepatitis A virus cellular receptor 2 [HAVCR2], and lymphocyte activating gene 3 [LAG3]) in different subgroups were estimated and these results are shown in Fig. 3A-E and Additional file 4: Figure S3A-G. Relative to Cluster 1, the PD-1, PD-L1, IDO1, HAVCR2, and LAG3 expression levels were all significantly higher in Cluster 2 (all *p* < 0.05). This suggested that Cluster 2 patients might benefit more from treatment with immune checkpoint inhibitors.

Further analysis indicated that LINC02084, PCED1B-AS1, LINC01857, LINC00861, LINC02381, and LINC01679 were positively correlated with the expression of PD-1, while STAG3L5P-PVRIG2P-PILRB, NCBP2-AS1, WARS2-AS1, and LENG8-AS1 were negatively correlated with the expressions of PD-1 (Fig. 3F). SNHG26, NSMCE1 − DT, LINC02084, PCED1B-AS1, MYOSLID, LINC02428, ATP2B1 − AS1, LINC01857, LINC00861, LINC02381, PRKAR1B − AS2, MRPS9 − AS1, and LINC01679 were positively correlated with the expression of PD-L1, while STAG3L5P-PVRIG2P-PILRB and LENG8-AS1 were negatively correlated with the expressions of PD-L1 (Fig. 3G). Moreover, the findings from the ESTIMATE analysis suggested that the immune (*p* < 0.001), stromal (*p* < 0.05), and estimate (*p* < 0.01) scores in Cluster 2 were markedly higher than those in Cluster 1 (Fig. 3H-J). Finally, we evaluated the abundances of 22 distinct kinds of immune cells between the two subtypes and found no differences (all *p* > 0.05, Fig. 3K). This suggested that the composition of immune cells in Cluster 2 was not significantly different from that in Cluster 1; however, the number of infiltrating immune cells and stromal cells was higher, and the tumor purity was lower in Cluster 2 than those in Cluster 1.

### Gene set enrichment analysis for clusters 1 and 2

**Fig. 4:**
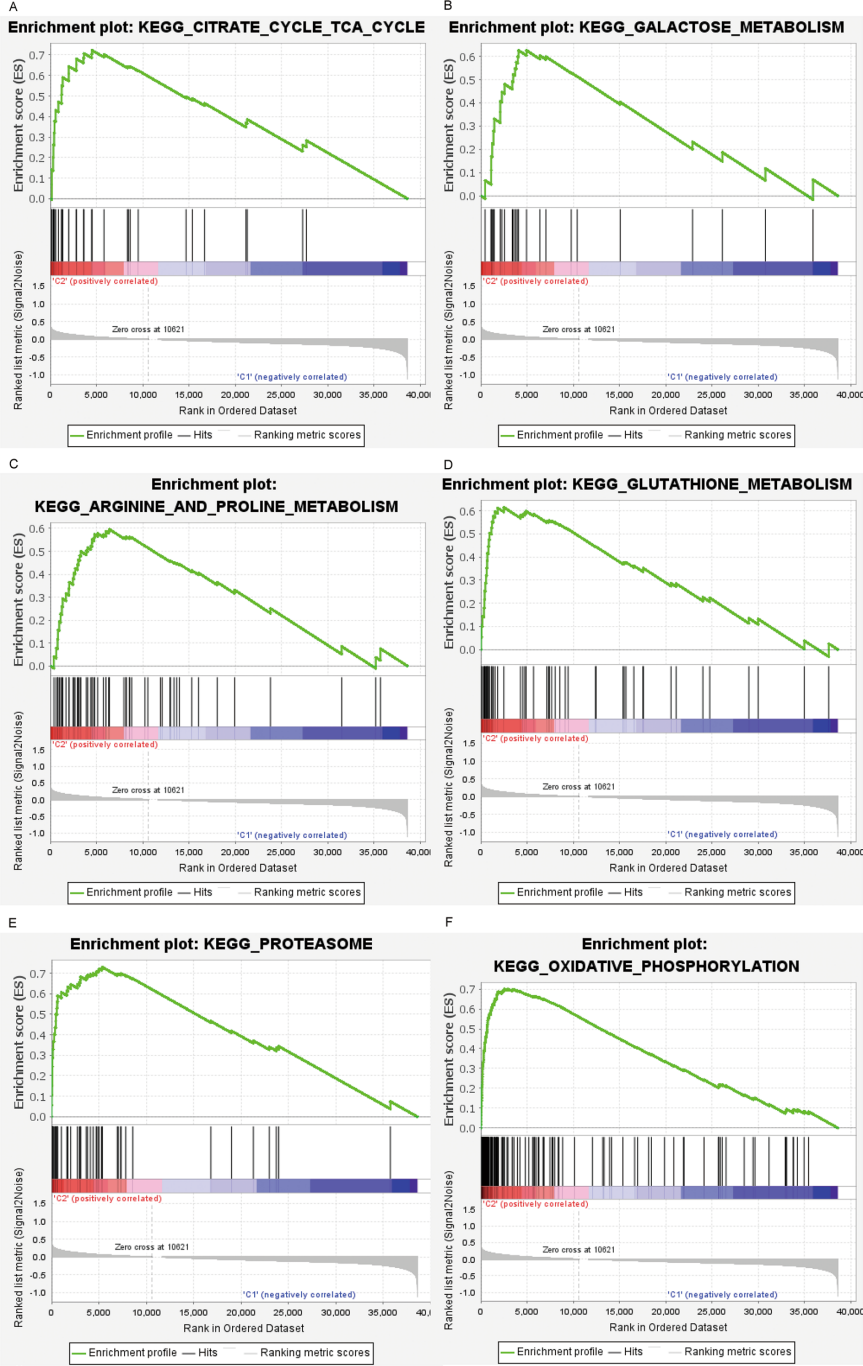
Gene set enrichment analysis in Clusters 1 and 2 on the basis of the KEGG pathways. The pathways related to **A** citrate cycle and TCA cycle, **B** galactose metabolism, **C** arginine and proline metabolism, **D** glutathione metabolism, **E** proteasome metabolism, and **F** oxidative phosphorylation are enriched in Cluster 2. KEGG, Kyoto Encyclopedia of Genes and Genomes; TCA, tricarboxylic acid cycle

To understand the mechanisms underlying prognostic and immunophenotypic differences between Clusters 1 and 2, we performed GSEA. The pathways related to citrate cycle and tricarboxylic acid cycle (TCA) cycle (*p* = 0.010), galactose metabolism (*p* = 0.002), proline and arginine metabolism (*p* < 0.001), glutathione metabolism (*p* = 0.002), proteasome (*p* = 0.010), and oxidative phosphorylation (*p* < 0.001) were considerably enriched in Cluster 2 (Fig. 4A–F; Additional file 5: Table S2). Previous results showed that Cluster 2 patients had better survival, indicating that the signaling pathway enriched in Cluster 2 might be involved in the inhibition of the occurrence and development of colon cancer.

### Construction of a lipid metabolism-LncRM for predicting survival

**Fig. 5:**
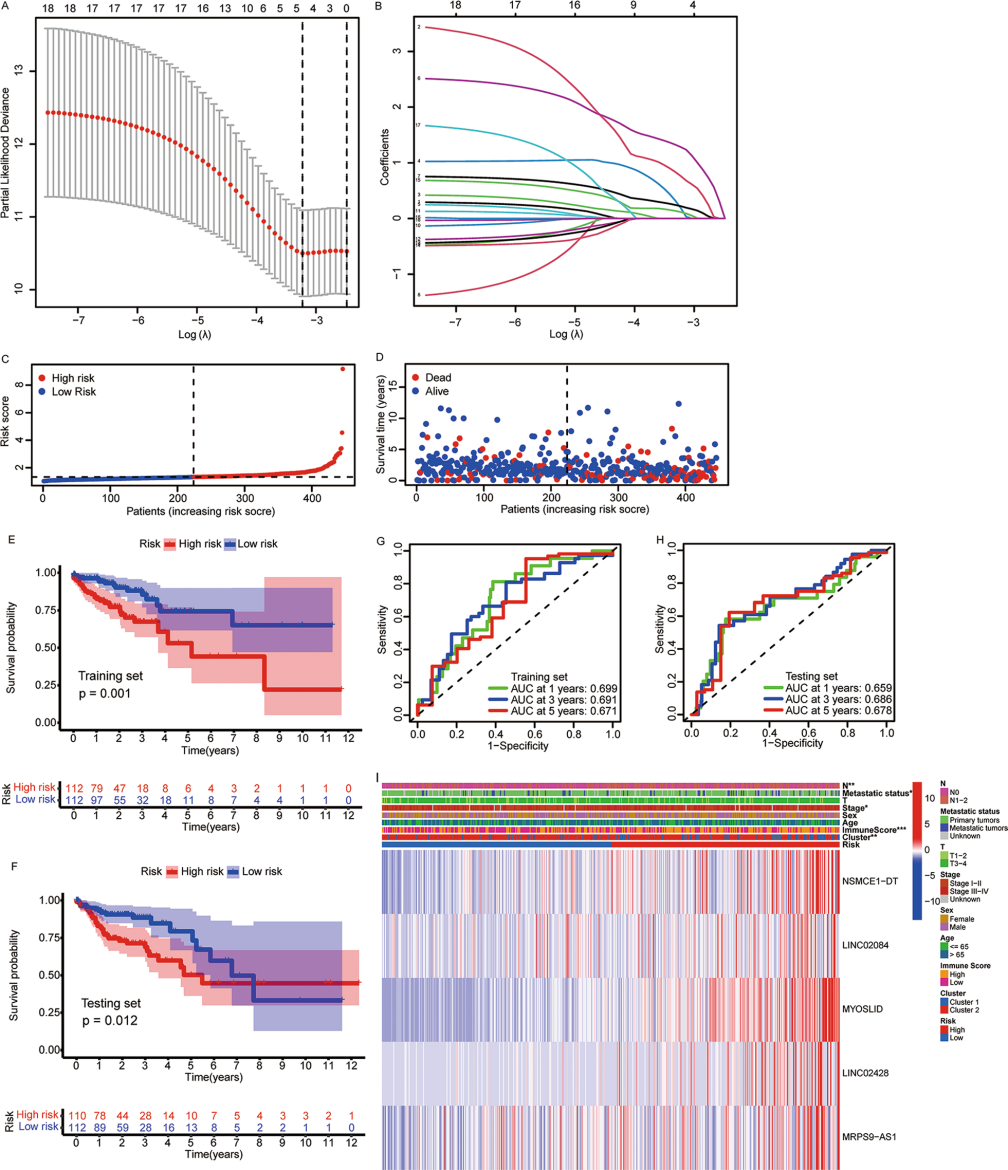
Construction and evaluation of the lipid metabolism-lncRNA prognostic model for colon cancer. **A**, **B** LASSO regression analysis of lipid metabolism-related lncRNAs that are most significantly correlated with OS. **C** Risk score and **D** survival status of each patient. OS of patients in high- and low-risk groups in **E** training and **F** testing cohorts. ROC analysis for risk scores in **G** training and **H** testing cohorts. **I** Expressions of five prognostic lipid metabolism-related lncRNAs in different risk groups. LASSO, least absolute shrinkage and selection operator; OS, overall survival; ROC, receiver operating characteristic. *p < 0.05, **p < 0.01, and ***p < 0.001

For an accurate prediction of colon cancer prognoses, five lncRNAs were filtered out by LASSO regression from among the 18 lipid metabolism-related prognostic lncRNAs (Fig. 5A, 5B). The patients were categorized into low- and high-risk groups predicated on the mean risk score (Fig. 5C, 5D). In both training (*p* = 0.001) and testing cohorts (*p* = 0.012), high-risk patients exhibited considerably reduced OS duration in contrast with that in the low-risk group (Fig. 5E, 5F). In addition, the ROC curves for OS at 1, 3, and 5 years in the training and testing cohorts are illustrated in Figs. 5G and 5H, respectively. As depicted in Fig. 5I, the high-risk patients showed elevated levels of NSMCE1-DT, LINC02084, MYOSLID, LINC02428, and MRPS9-AS1 expressions (all *p* < 0.05). This is consistent with the results shown in Fig. 1B, indicating that these five lncRNAs were all related to poor prognosis.

According to the R package “ImmuneSubtypeClassifier,” tumor samples were divided into five immune subtypes: C1 (wound healing), C2 (IFN-gamma dominant), C3 (inflammatory), C4 (lymphocyte depleted), and C6 (TGF-b dominant). However, C5 (immunologically quiet) subtype was not present. The relationships between the five lncRNAs in the prognostic model and immune subtypes are shown in Additional file 6: Figure S4. Only LINC02084 was differentially expressed among the different immune subtypes (*p* < 0.001), indicating that the expression level of LINC02084 was related to immune subtypes. In addition, the relationships between lncRNAs and the four molecular subtypes of colon cancer are shown in Additional file 7: Figure S5. In addition to MRPS9-AS1, the expression levels of the remaining four lncRNAs were associated with the molecular subtypes (all *p* < 0.01).

### Association between clinical characteristics and risk score

**Fig. 6:**
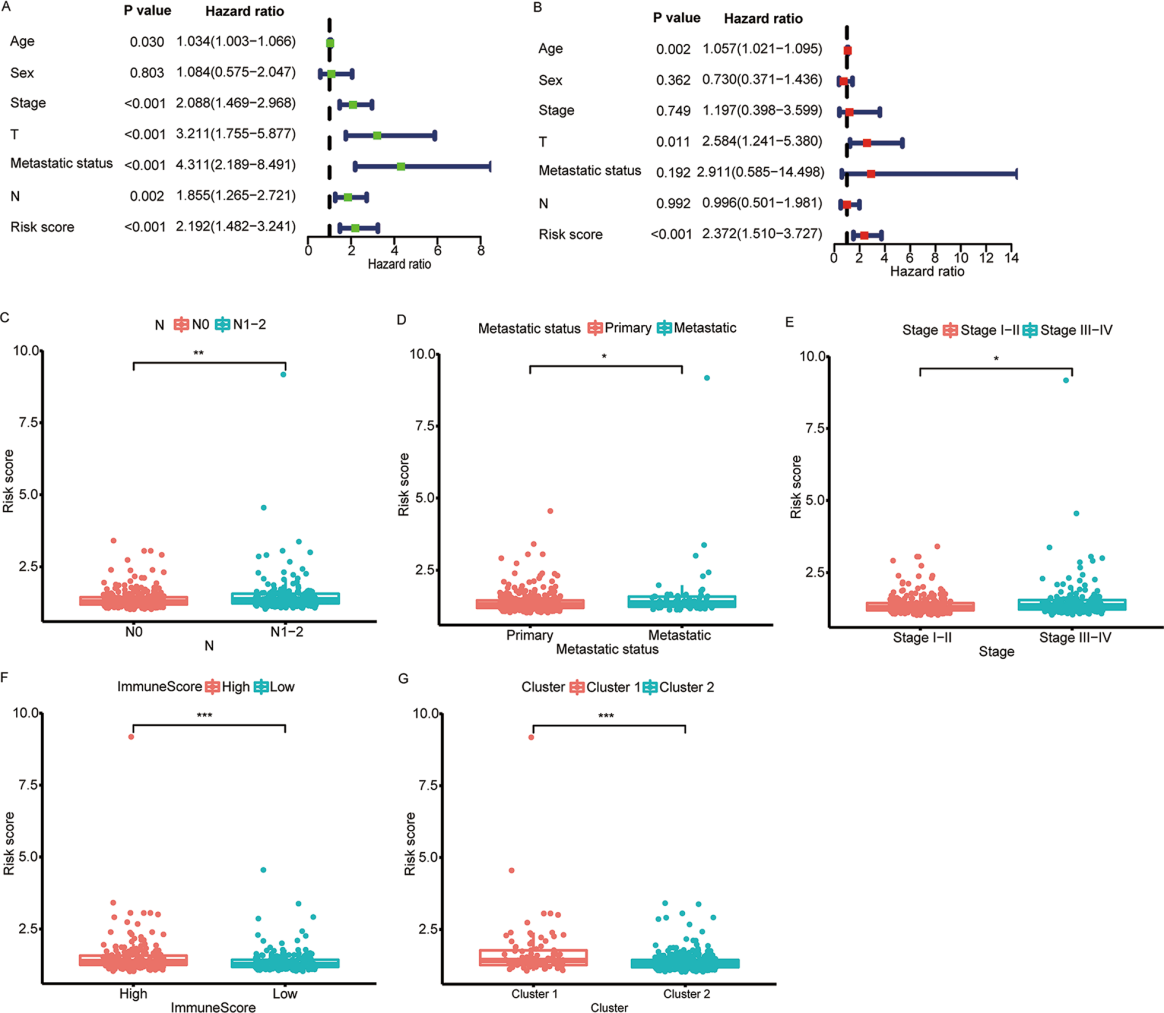
Relationship among risk scores and clinical characteristics. Forest plots based on **A** univariate and **B** multivariate Cox regression analysis for OS. Correlations of risk scores and **C** N stage, **D** metastatic status, **E** total stage, **F** immune score, and **G** Clusters. OS, overall survival. *p < 0.05, **p < 0.01, and ***p < 0.001

Figures 6A and 6B indicated that risk score independently served as a predictor of OS (both *p* < 0.001). In addition, high-risk scores had a high likelihood of being associated with distant metastases, higher N staging, total staging, and immunity scores, and a high likelihood of belonging to the Cluster 1 subtype (all *p* < 0.05, Fig. 6C-G and Additional file 8: Figure S6). This may be part of the reason for the worse prognosis of high-risk patients.

### Correlation between lipid metabolism-LncRM and tumor microenvironment

**Fig. 7:**
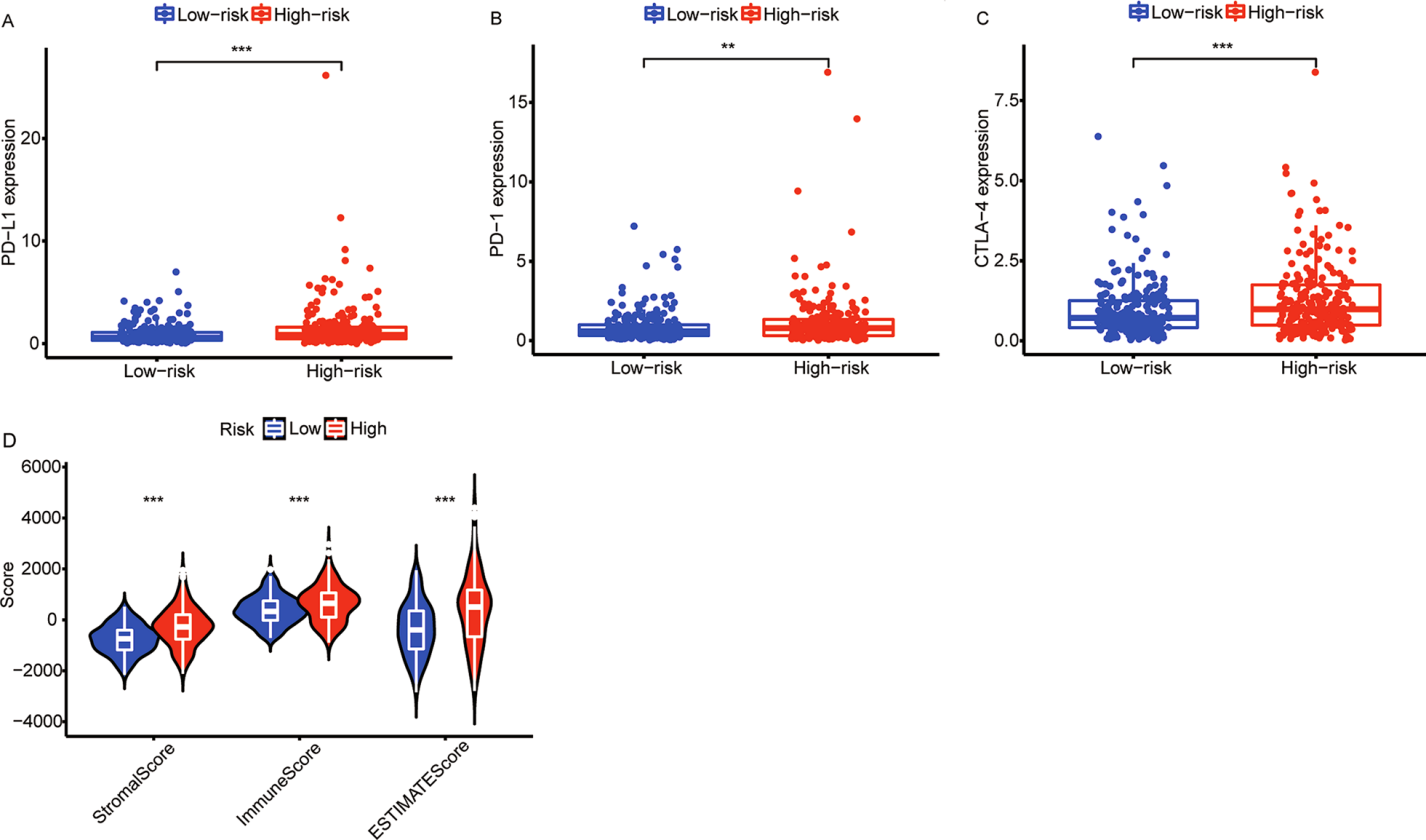
Correlation of risk score and tumor microenvironment. The expressions of **A** PD-L1, **B** PD-1, and **C** CTLA-4 in different risk groups. **D** Tumor microenvironment scores for different risk groups. PD-1, programmed cell death 1; PD-L1, programmed cell death ligand 1; CTLA-4, cytotoxic T lymphocyte antigen-4. **p < 0.01, and ***p < 0.001

**Fig. 8:**
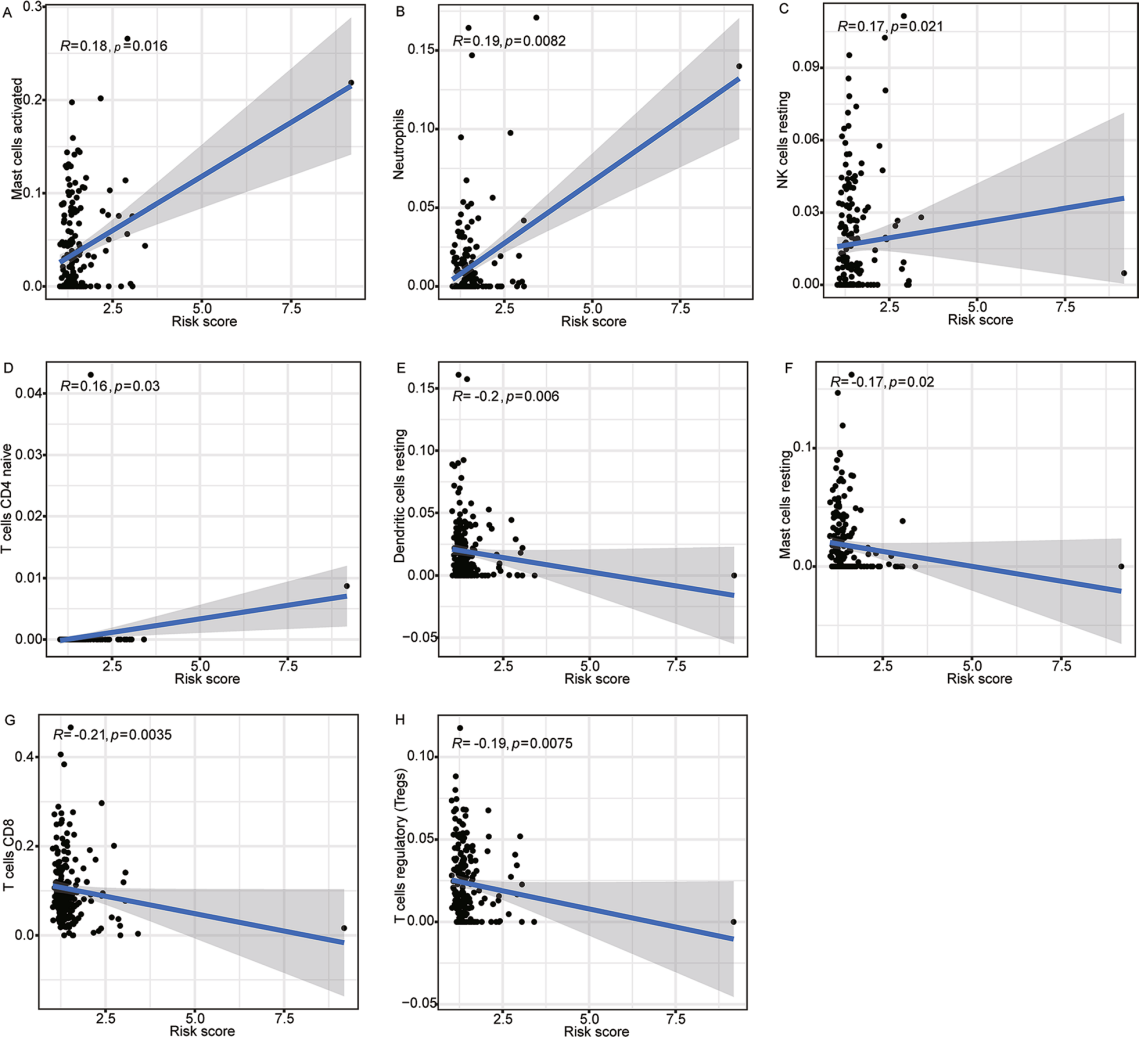
Relationship among risk scores and immune cell infiltrates. Correlations of risk scores and **A** mast cells activated, **B** neutrophils, **C** NK cells resting, **D** T cells CD4 naive, **E** dendritic cells resting, **F** mast cells resting, **G** T cells CD8, and **H** T cells regulatory

As shown in Fig. 7A-C and Additional file 4: Figure S3H-N, as opposed to the low-risk colon cancer patients, the high-risk cases showed higher expressions of CTLA-4 (*p* < 0.001), PD-1 (*p* < 0.01), PD-L1 (*p* < 0.001), IDO1 (*p* < 0.05), IDO2 (*p* < 0.05), HAVCR2 (*p* < 0.001), and LAG3 (*p* < 0.05). The estimate, stromal, and immune scores were elevated in the high-risk group in comparison to the low-risk subgroup (all *p* < 0.001, Fig. 7D). The abundances of immune cells estimated by CIBERSORT analysis are depicted in Fig. 8A–H. Mast cells activated, CD4 + T cells naive, NK cells resting, and Neutrophils activated were positively correlated with risk scores (all *p* < 0.05), while CD8 + T cells, T regulatory cells, mast cells resting, and dendritic cells resting were found to have a negative link to risk scores (all *p* < 0.05). This phenomenon shows that the higher the risk score, the worse the anti-tumor immune ability.

## Discussion

In the present study, 18 lipid metabolism-related prognostic lncRNAs were analyzed. By consensus clustering, colon cancer patients were classified into two distinct categories. In Cluster 2, the survival time of patients was prolonged, and the expressions of PD-1, PD-L1, IDO1, HAVCR2, and LAG3 were elevated. In addition, metabolic-related pathways were highly enriched in Cluster 2. Five lipid metabolism-related lncRNAs most significantly correlated with prognosis were selected using LASSO analysis to construct the lipid metabolism-LncRM. Using multivariate analysis, we identified the risk score as an independent and adverse predictor for OS. Moreover, patients with distinct risk scores exhibited varying survival status, clinical characteristics, and immune cell infiltration. We confirmed that lipid metabolism-related lncRNAs may serve as a signature for predicting survival and is a potential immunotherapeutic target for patients with colon cancer.

Energy metabolism reprogramming is an important feature of tumors [21, 22]. The imbalance among oncogenic pathways interferes with the synthesis and metabolism of various substances, including glucose, glutamine, and fatty acids, thereby promoting the growth of tumor cells [23–25]. To date, studies on the association of lncRNA expression with lipid metabolism in colon cancer are scarcely reported. Therefore, it is necessary to investigate lipid metabolism-related lncRNAs using “big data”. Christensen et al. [26] show that the lncRNA SNHG16 performs an important function in the transcription of genes related to lipid metabolism and can target up to 26 microRNA families. Yurui et al. [27] have constructed a competing endogenous RNA (ceRNA) network related to fatty acid metabolism. They report that lipid metabolism-related lncRNAs can potentially affect the prognoses of colon cancer patients. Metabolic reprogramming varies between different cell subtypes. In this study, the population was divided into two subgroups based on lipid metabolism-related lncRNAs Cluster recognition. Different subgroups showed varying survival times and risks of distant metastases. Further, significant differences were found in glucose metabolism and lipid metabolism pathways. Therefore, the study of lipid metabolism-related lncRNAs may help to further classify patients and provide individualized treatment options.

To better predict the prognoses of patients, we finally identified five prognostic-associated lipid metabolism-related lncRNAs, namely NSMCE1-DT, LINC02084, MYOSLID, LINC02428, and MRPS9-AS1. LINC02084 has been previously linked to the prognoses of patients with hepatocellular carcinoma [28]. MYOSLID is involved in the advancement of head and neck squamous cell carcinomas (HNSCC) and osteosarcoma, and its mechanism of action is by promoting RAB13 expression by sponging microRNA-1286 or promoting invasion and metastases by regulating a part of the epithelial-mesenchymal transformation [29, 30]. As far as we know, this is the first research on the relationship of NSMCE1-DT, LINC02428, and MRPS9-AS1 with tumor prognosis. The underlying mechanisms that affect patient survival warrant further investigations.

In addition, based on the lipid metabolism-LncRM, we categorized the patients into low- and high-risk groups and compared the survival, clinicopathological features, immune cell infiltrates, and the expressions of immune checkpoints between the two groups. The high-risk group was found to show higher staging and poorer survival. Further analysis demonstrated that low infiltration levels of CD8 + T cells and dendritic cells resting, and elevated expression levels of immune checkpoints in these patients, thereby suggesting that immunotherapeutic intervention for patients in the high-risk group might improve tumor control.

Currently, immunotherapy is an emerging and dynamic area in cancer treatment, including, but not limited to, cancer vaccines, oncolytic viruses, and immune checkpoint inhibitors [31–33]. CTLA-4, PD-1, and PD-L1 are responsible for the immunotherapeutic responses in patients with colon cancer. Fiegle et al. [34] demonstrate the association of PD-1 and CTLA-4 overexpression with a worse OS and disease-free survival. In the CheckMate-142 (NCT02060188) study, the effects of nivolumab monotherapy on 74 patients with microsatellite instability-high (MSI-H) or mismatch repair deficiency (dMMR) metastatic colorectal carcinoma (mCRC) were reported, wherein the estimated rates at 12 months were 50% for progression-free survival and 73% for OS [35]. In addition, nivolumab plus ipilimumab in the treatment of MSI-H MCRC showed a disease control rate of 80%, and the objective remission rate was 55% (95% CI 45.2–63.8) [36]. Therefore, although high-risk patients identified through the prognostic model established in this study showed poor survival, they may potentially benefit more from therapy using immune checkpoint inhibitors.

In theory, PD-L1 is a viable marker of anti-PD-1 immunotherapy. It is reported that mCRC patients who responded to PD-1 inhibitors show dMMR status or PD-L1 up-regulation in tumor cells [37, 38]. Further studies show that PD-L1 overexpression is related to better clinical prognoses. This may be due to compensatory regulation of this marker, thereby threatening the tumor with an aggressive immune response [39]. Therefore, PD-L1 expression and dMMR genotype are considered possible markers of responses to PD-1 inhibitors. However, some studies report that the level of PD-L1 expression is not linked to the treatment efficacy in CRC patients [40, 41]. In addition to PD-L1, earlier studies have also illustrated that the existence of tumor-infiltrating lymphocytes in the TME is closely related to a better response rate toward anti-tumor immunotherapy [42–44]. In our study, the expression level of PD-L1 was discovered to be higher in the high-risk subgroup, accompanied by significant infiltrations of immune cells in the TME, indicating that the high-risk group could be highly suited for immunotherapeutic treatments.

Using lipid metabolism-related lncRNAs, herein, we have provided a new perspective for prognostic prediction and treatment formulation for patients with colon cancer. Nevertheless, there are several drawbacks to the research. Our results were based entirely on bioinformatic analyses, and more trials are required to confirm the mechanism behind lncRNA action. Further, prospective clinical data are needed to verify the prognostic and therapeutic values of lipid metabolism-LncRM, accounting for the inherent deficiencies of this retrospective analysis.

## Conclusions

In conclusion, we confirmed the association of lipid metabolism-related lncRNAs in colon cancer with the prognoses of patients, the expressions of immune checkpoints, and the infiltrations of different immune cells in the TME. These lncRNAs could function as new potential targets for treating colon cancer.

## Supplementary Information

**Additional file 1: Figure S1.** Flow chart of the search protocol and study design. TCGA, The Cancer Genome Atlas; lncRNAs, long non-coding RNAs; LASSO, Least Absolute Shrinkage Selection Operator; GSEA, gene set enrichment analysis.

**Additional file 2: Table S1.** Genes related to lipid metabolism.

**Additional file 3: Figure S2.** Consensus clustering based on prognostic lipid metabolism-related lncRNAs. **A** Consensus clustering cumulative distribution function (CDF) for k = 2 to 9. **B** Relative changes in the areas under the CDF curve for k = 2 to 9. Consensus matrix similarity for **C** 2, **D** 3, **E** 4, and **F** 5 clusters.

**Additional file 4: Figure S3.** Association of immune checkpoints with Clusters and prognostic risk groups. The expression of immune checkpoints **A** PD-1, **B** PD-L1, **C** CTLA-4, **D** IDO1, **E** IDO2, **F** HAVCR2, and **G** LAG3 in Clusters 1 and 2. The expressions of H PD-L1, I PD-1, J CTLA-4, K IDO1, L IDO2, M HAVCR2, and N LAG3 in different risk groups. PD-1, programmed cell death 1; PD-L1, programmed cell death ligand 1; CTLA-4, cytotoxic T lymphocyte antigen-4; IDO1, indoleamine 2,3-dioxygenase 1; IDO2, indoleamine 2,3-dioxygenase 2; HAVCR2, hepatitis A virus cellular receptor 2; LAG3, lymphocyte activating gene 3. ns, not significant; *p < 0.05, **p < 0.01, and ***p < 0.001.

**Additional file 5: Table S2.** The enriched pathways of the Cluster 1 and 2.

**Additional file 6: Figure S4.** Association of each lncRNA in the identified signature with the immune subtypes. The expression of lncRNA **A** NSMCE1-DT, **B** LINC02084, **C** MYOSLID, **D** LINC02428, and **E** MRPS9-AS1 in different immune subtypes. -, not significant, *p < 0.05, and ***p < 0.001.

**Additional file 7: Figure S5.** Association of each lncRNA in the identified signature with the molecular subtypes. The expression of lncRNA **A** NSMCE1-DT, **B** LINC02084, **C** MYOSLID, **D** LINC02428, and **E** MRPS9-AS1 in different molecular subtypes. MSI, microsatellite instability; CIN, chromosomal instability; GS, genome-stable; HM-SNV, hypermutated single nucleotide variants. -, not significant; *p < 0.05, **p < 0.01, and ***p < 0.001.

**Additional file 8: Figure S6.** Relationship among risk scores and clinical characteristics. Correlations of risk scores and **A** age, **B** sex, **C** T stage, **D** N stage, **E** metastatic status, **F** total stage, **G** immune score, and **H** Clusters. ns, not significant; *p < 0.05, **p < 0.01, and ***p < 0.001.

## Data Availability

Publicly available datasets were analyzed in this study. This data can be found here: http://cancergenome.nih.gov/.

## Abbreviations

OS: Overall survival
lncRNAs: Long non-coding RNAs
TCGA: The cancer genome atlas
LASSO: The least absolute shrinkage selection operator
KEGG: The kyoto encyclopedia of genes and genomes
GSEA: Gene set enrichment analysis
TME: Tumor microenvironment
lipid metabolism-LncRM: Prognostic model based on lipid metabolism-related lncRNAs
ROC: Receiver operating characteristic
CI: Confidence interval
PD-1: Programmed cell death 1
PD-L1: Programmed cell death ligand 1
CTLA-4: Cytotoxic T lymphocyte antigen-4
IDO1: Indoleamine 2,3-dioxygenase 1
IDO2: Indoleamine 2,3-dioxygenase 2
HAVCR2: Hepatitis A virus cellular receptor 2
LAG3: Lymphocyte activating gene 3
TCA: Tricarboxylic acid cycle
ceRNA: Competing endogenous RNA
HNSCC: Head and neck squamous cell carcinomas
MSI-H: Microsatellite instability-high
dMMR: Mismatch repair deficiency
mCRC: Metastatic colorectal carcinoma
