# Supplementary figures and images for: Role of a lipid metabolism-related lncRNA signature in risk stratification and immune microenvironment for colon cancer
Source: BMC Med Genomics. 2022 Oct 21;15:221. doi: 10.1186/s12920-022-01369-8 (PMC9590147; doi:10.1186/s12920-022-01369-8)

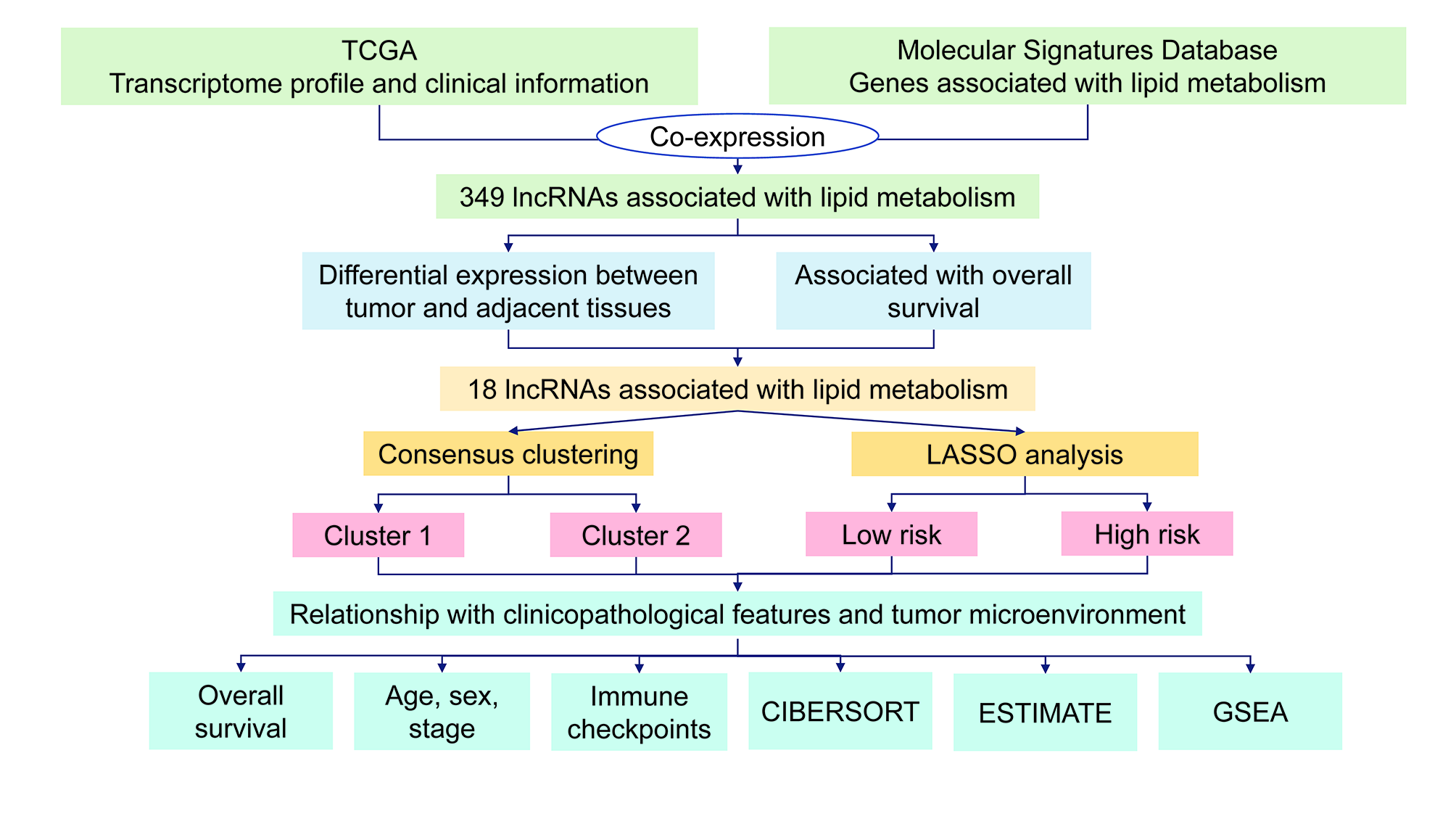

Supplement: Supplementary file 1 — Additional file 1: Figure S1. Flow chart of the search protocol and study design. TCGA, The Cancer Genome Atlas; lncRNAs, long non-coding RNAs; LASSO, Least Absolute Shrinkage Selection Operator; GSEA, gene set enrichment analysis. [file 12920_2022_1369_MOESM1_ESM.tiff]

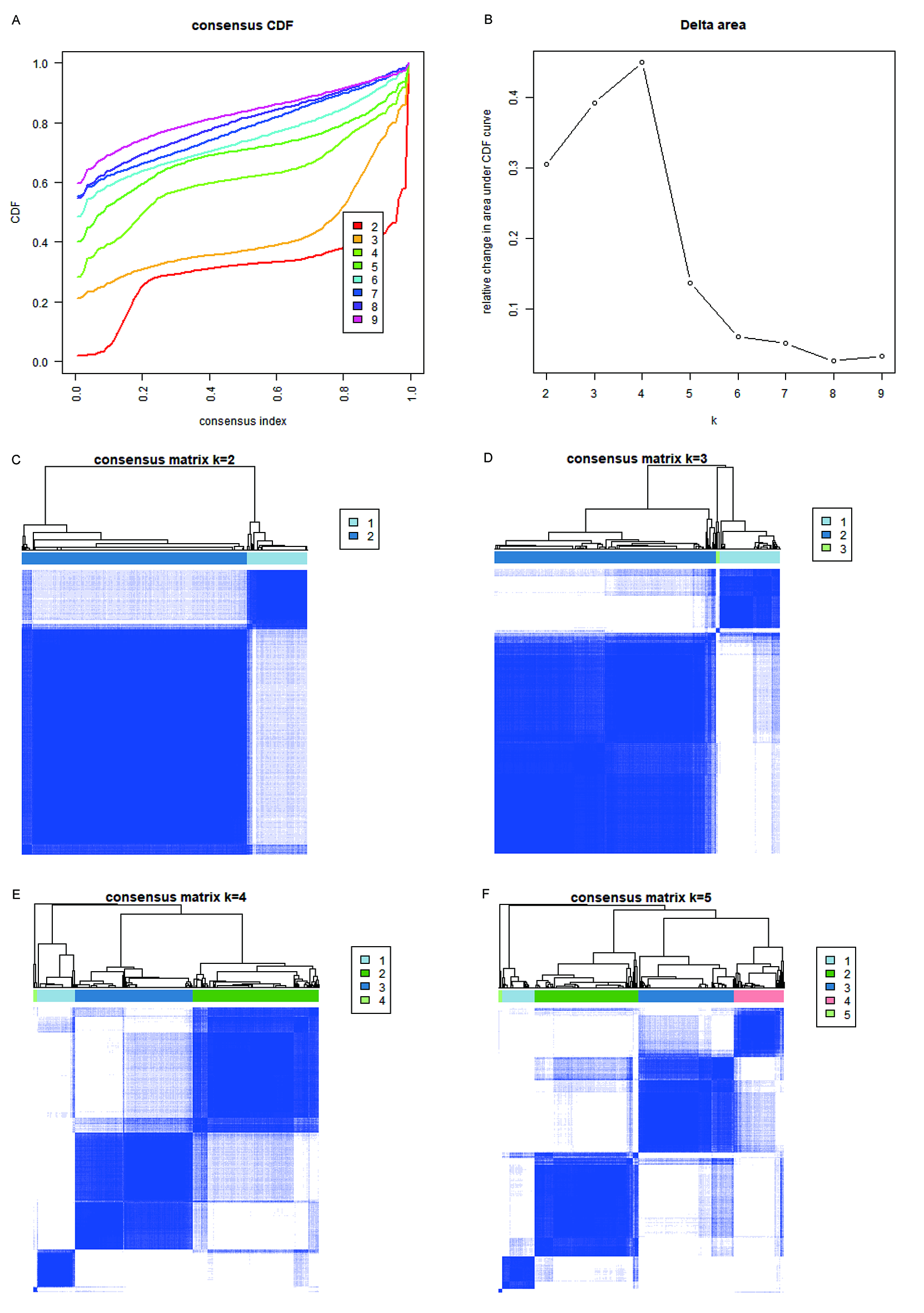

Supplement: Supplementary file 3 — Additional file 3: Figure S2. Consensus clustering based on prognostic lipid metabolism-related lncRNAs. A Consensus clustering cumulative distribution function (CDF) for k = 2 to 9. B Relative changes in the areas under the CDF curve for k = 2 to 9. Consensus matrix similarity for C 2, D 3, E 4, and F 5 clusters. [file 12920_2022_1369_MOESM3_ESM.tiff]

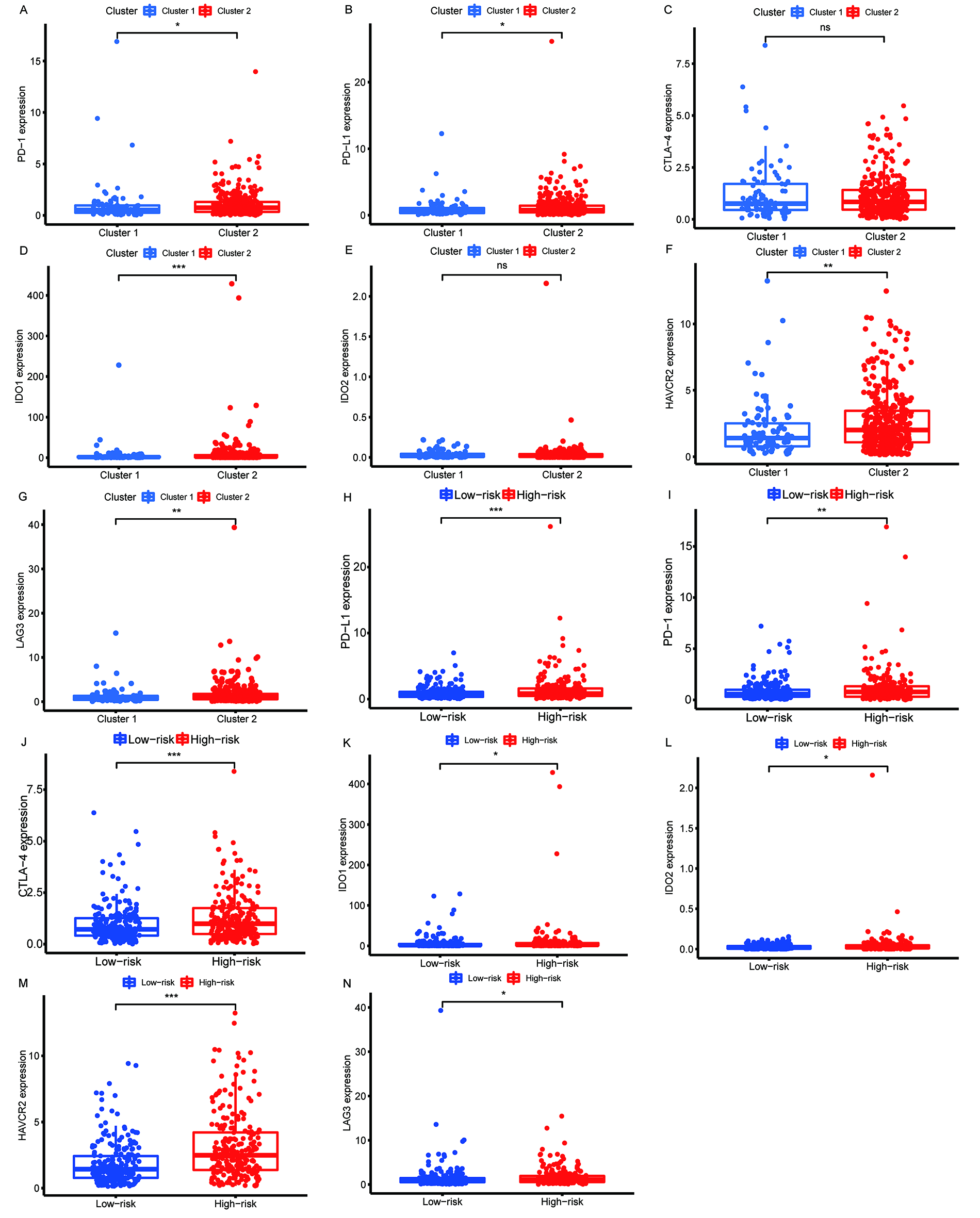

Supplement: Supplementary file 4 — Additional file 4: Figure S3. Association of immune checkpoints with Clusters and prognostic risk groups. The expression of immune checkpoints A PD-1, B PD-L1, C CTLA-4, D IDO1, E IDO2, F HAVCR2, and G LAG3 in Clusters 1 and 2. The expressions of H PD-L1, I PD-1, J CTLA-4, K IDO1, L IDO2, M HAVCR2, and N LAG3 in different risk groups. PD-1, programmed cell death 1; PD-L1, programmed cell death ligand 1; CTLA-4, cytotoxic T lymphocyte antigen-4; IDO1, indoleamine 2,3-dioxygenase 1; IDO2, indoleamine 2,3-dioxygenase 2; HAVCR2, hepatitis A virus cellular receptor 2; LAG3, lymphocyte activating gene 3. ns, not significant; *p < 0.05, **p < 0.01, and ***p < 0.001. [file 12920_2022_1369_MOESM4_ESM.tiff]

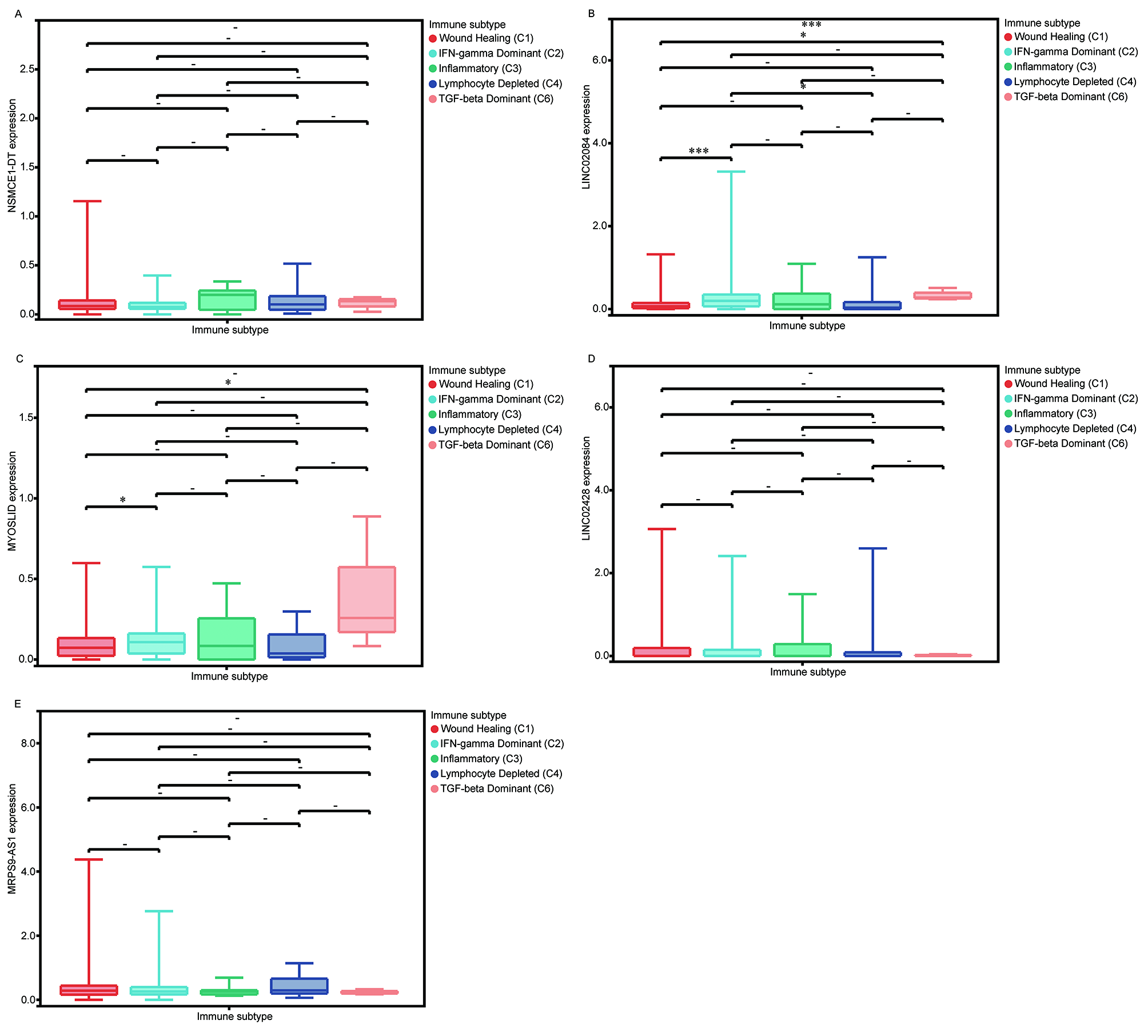

Supplement: Supplementary file 6 — Additional file 6: Figure S4. Association of each lncRNA in the identified signature with the immune subtypes. The expression of lncRNA A NSMCE1-DT, B LINC02084, C MYOSLID, D LINC02428, and E MRPS9-AS1 in different immune subtypes. -, not significant, *p < 0.05, and ***p < 0.001. [file 12920_2022_1369_MOESM6_ESM.tiff]

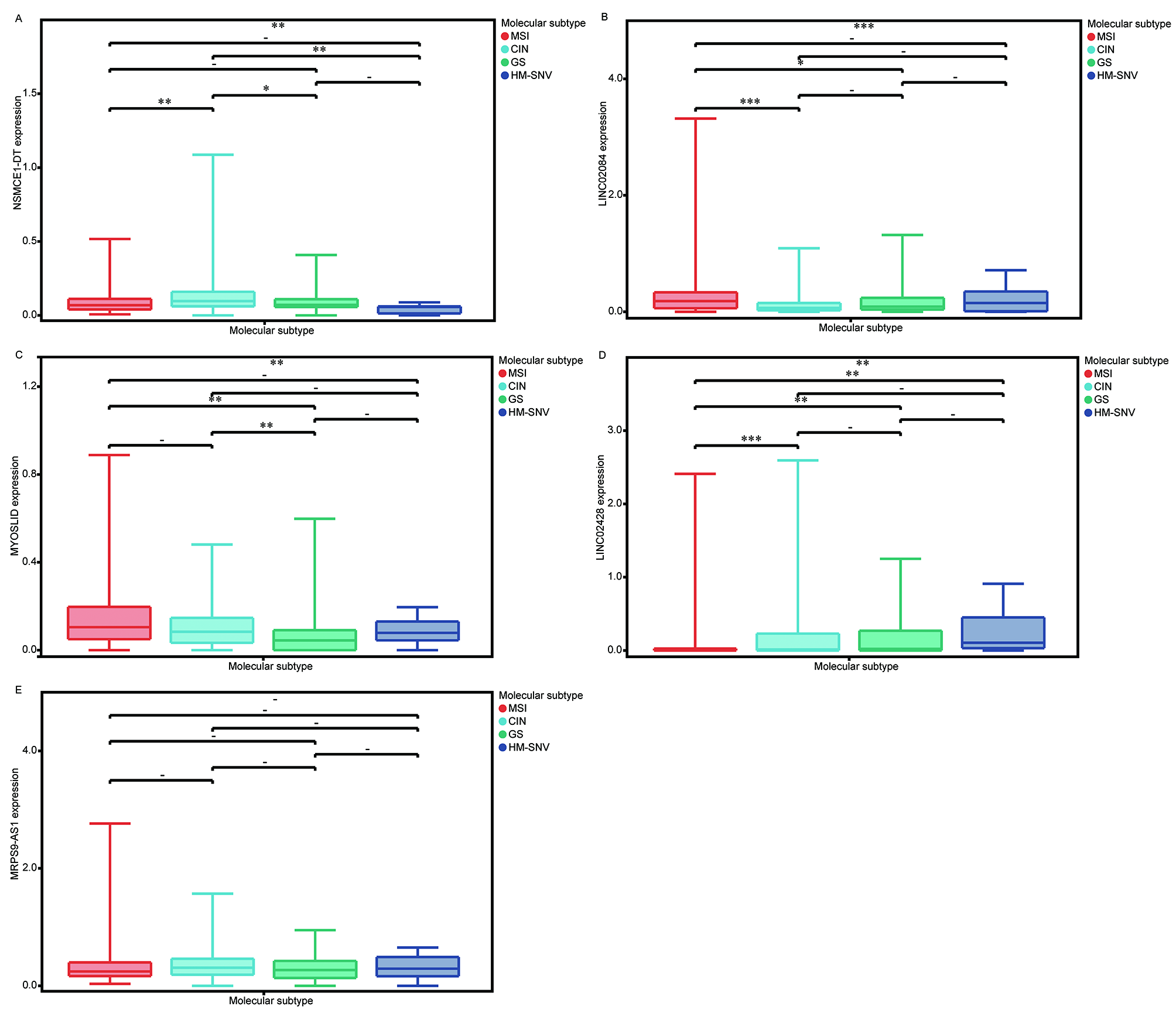

Supplement: Supplementary file 7 — Additional file 7: Figure S5. Association of each lncRNA in the identified signature with the molecular subtypes. The expression of lncRNA A NSMCE1-DT, B LINC02084, C MYOSLID, D LINC02428, and E MRPS9-AS1 in different molecular subtypes. MSI, microsatellite instability; CIN, chromosomal instability; GS, genome-stable; HM-SNV, hypermutated single nucleotide variants. -, not significant; *p < 0.05, **p < 0.01, and ***p < 0.001. [file 12920_2022_1369_MOESM7_ESM.tiff]

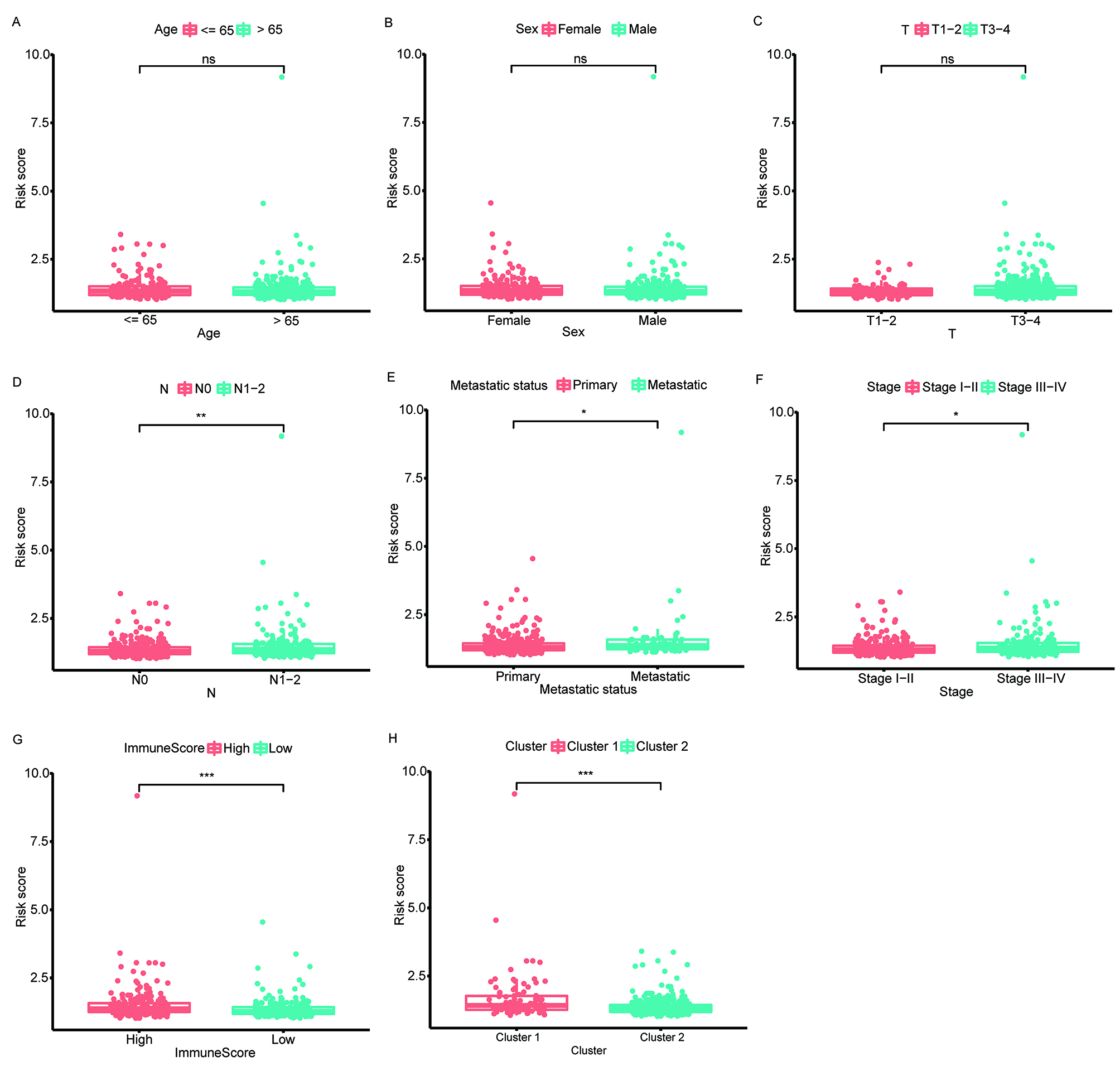

Supplement: Supplementary file 8 — Additional file 8: Figure S6. Relationship among risk scores and clinical characteristics. Correlations of risk scores and A age, B sex, C T stage, D N stage, E metastatic status, F total stage, G immune score, and H Clusters. ns, not significant; *p < 0.05, **p < 0.01, and ***p < 0.001. [file 12920_2022_1369_MOESM8_ESM.tiff]
